# CRISPR/dCas9-induced upregulation of endogenous apolipoprotein A1 and paraoxonase 1 genes reduces the aortic lipid deposits in apoE^−/−^ mice

**DOI:** 10.1186/s43556-026-00580-8

**Published:** 2026-09-18

**Authors:** Laura Toma, Teodora Barbălată, Jessica Isabela Cătălina Hărătău, Gabriela Maria Sanda, Elena Valeria Fuior, Ioana Mădălina Fenyo, Loredan Ştefan Niculescu, Laura-Maria Dăian, Shlomo Sasson, Anca Volumnia Sima, Camelia Sorina Stancu

**Affiliations:** 1 Lipidomics Department, Institute of Cellular Biology and Pathology “, Institute of Cellular Biology and Pathology “Nicolae Simionescu” of the Romanian Academy, 8, B.P. Haşdeu Street, Bucharest, 050568 Romania; 2 Gene Regulation and Molecular Therapies Laboratory, Institute of Cellular Biology and Pathology “Nicolae Simionescu” of the Romanian Academy, 8, B.P. Haşdeu Street, Bucharest, 050568 Romania; 3 BetaUpreg Research Group, Institute of Cellular Biology and Pathology “Nicolae Simionescu” of the Romanian Academy, 8, B.P. Haşdeu Street, Bucharest, 050568 Romania; 4 Department of Pharmacology, Institute for Drug Research, Faculty of Medicine, The Hebrew University of Jerusalem, Jerusalem, 9112002 Israel

**Keywords:** Apolipoprotein A1, CRISPR/dCas9, Endothelial cells, Hepatocytes, Lipid deposits, Paraoxonase 1

## Abstract

High-density lipoproteins (HDL) are essential to alleviate the progression of atherosclerosis by mediating reverse-cholesterol transport, antioxidant and anti-inflammatory effects. We aimed to enhance the expression of endogenous HDL components, apolipoprotein A1 (APOA1) and antioxidant enzyme paraoxonase 1 (PON1), and to investigate their athero-protective effects. The CRISPR/dCas9 technology was used to activate the transcription of endogenous *APOA1/PON1* in human hepatocytes (Huh7 line) and *Apoa1*/*Pon1* in apoE^−/−^ mice. The expression of *APOA1/PON1* genes was successfully upregulated in hepatocytes, and their proteins were secreted in the culture medium in the presence/absence of tumor necrosis factor-α (TNFα). APOA1-rich Huh7-derived conditioned medium exerted antioxidant and anti-inflammatory effects in TNFα-activated EA.hy926 endothelial cells. A single dose of the CRISPR/dCas9 plasmids i.v. injected in apoE^−/−^ mice increased the expression of hepatic *Apoa1/Pon1* and their serum levels up to four weeks. FPLC analysis showed that increased serum APOA1 was distributed between HDL, LDL, and in lipid-free form. These mice also exhibited high levels of hepatic, gallbladder and feces cholesterol, in part due to the upregulation of hepatic scavenger receptor class-B1, cholesterol 7-alpha-hydroxylase, and ATP-binding cassette sub-family-G-member-8 transporter. In apoE^−/−^ mice with upregulated *Apoa1/Pon1*, no increased inflammatory stress or innate immune activation were detected, while lipid peroxides were decreased in PON1 mice. Of major interest, the area of aortic lipid deposits was halved in the treated mice. Our findings demonstrate the successful upregulation of endogenous *Apoa1/Pon1* in apoE^−/−^ mice by using the CRISPR/dCas9 system, and highlight new mechanisms for APO1/PON1 anti-atherosclerotic action, explaining the reduction of aortic lipid deposits.

**Supplementary Information:**

The online version contains supplementary material available at https://doi.org/10.1186/s43556-026-00580-8.

## Introduction

Cardiovascular diseases (CVD) remain a worldwide menace, despite the advancement of therapeutic approaches. A critical early condition for the inception of atherosclerosis is the dysfunction of endothelial cells (EC) in blood vessels. Excessive oxidative and inflammatory stress favor increased transport of plasma proteins and lipoproteins in the subendothelium, and the adhesion and transmigration of blood monocytes due to an amplified expression of membrane adhesion molecules [1].

High density lipoproteins (HDL) play important anti-atherosclerotic roles by mediating reverse cholesterol transport (RCT), antioxidant, and anti-inflammatory effects [2, 3]. It is commonly accepted that the circulating levels of HDL-cholesterol (HDL-C) are inversely correlated with the cardiovascular risk [4–6]. Apolipoprotein A1 (APOA1) and paraoxonase 1 (PON1) are critical for HDL protective function due to their well-known role in regulating cholesterol transport and lipid metabolism, and in alleviating the oxidative and inflammatory stress [5, 6]. APOA1 and PON1 are expressed predominantly in the liver, and secreted in the circulation, being involved in HDL assembly and function [2, 7]. It has been established that APOA1 and PON1 levels are negatively correlated with atherosclerosis progression [8, 9]. Recently, in an in vitro study, Geh et al. [10] found that lipid-free, exchangeable APOA1 potently and competitively inhibits LDL binding to proteoglycans and thus can reduce LDL retention and lipid deposition in the arterial wall. Under oxidative stress conditions, both APOA1 and PON1 are prone to oxidation, which alters their structure and consequently their function [6, 7, 11]. Thus, APOA1 oxidation decreases cholesterol efflux [12] and fails to exert antioxidant and anti-inflammatory effects in activated EC [6, 8]. The oxidation of PON1 impairs the stability and activity of the enzyme and reduces its binding capacity to HDL [13]. The altered function of these proteins results in the appearance of dysfunctional and pro-atherogenic HDL [3, 6, 8]. Thus, it is accepted that elevated APOA1 or PON1 levels play a beneficial role in CVD patients [3, 6, 13]. Various studies show that HDL mimetics infusion protects CVD patients, but they require weekly administration [14, 15]. Therefore, further studies are needed to improve the therapeutic approach of enhancing APOA1 or PON1 levels over long periods of time to better protect and delay acute CVD events.

The CRISPR/Cas9 system has primarily been used to edit genome DNA sequences [16–18]. The powerful tool of CRISPR/deactivated Cas9 (dCas9) system, derived from CRISPR/Cas9 by inactivation of endonuclease Cas9, which can activate endogenous genes expression without cutting DNA has been less practiced and has generated interest for therapeutic applications due to its high efficiency and specificity [19].

The present study stemmed from the hypothesis that activation of transcription and translation of *APOA1* and *PON1* genes would exhibit protective effects and that this approach may translate into future development of effective anti-atherosclerotic therapy. This is a pioneering study on the outcome of CRISPR/dCas9-induced transcriptional activation of endogenous *APOA1* or *PON1* in cultured hepatocytes and of *Apoa1/Pon1* in apolipoprotein E-deficient (apoE^−/−^) mice, and on their effects in vascular EC function and on the area of aortic lipid-laden lesions.

## Results

### The transcription of endogenous APOA1 and PON1 is successfully activated in Huh7 hepatocytes by CRISPR/dCas9, under standard or inflammatory conditions

**Fig. 1:**
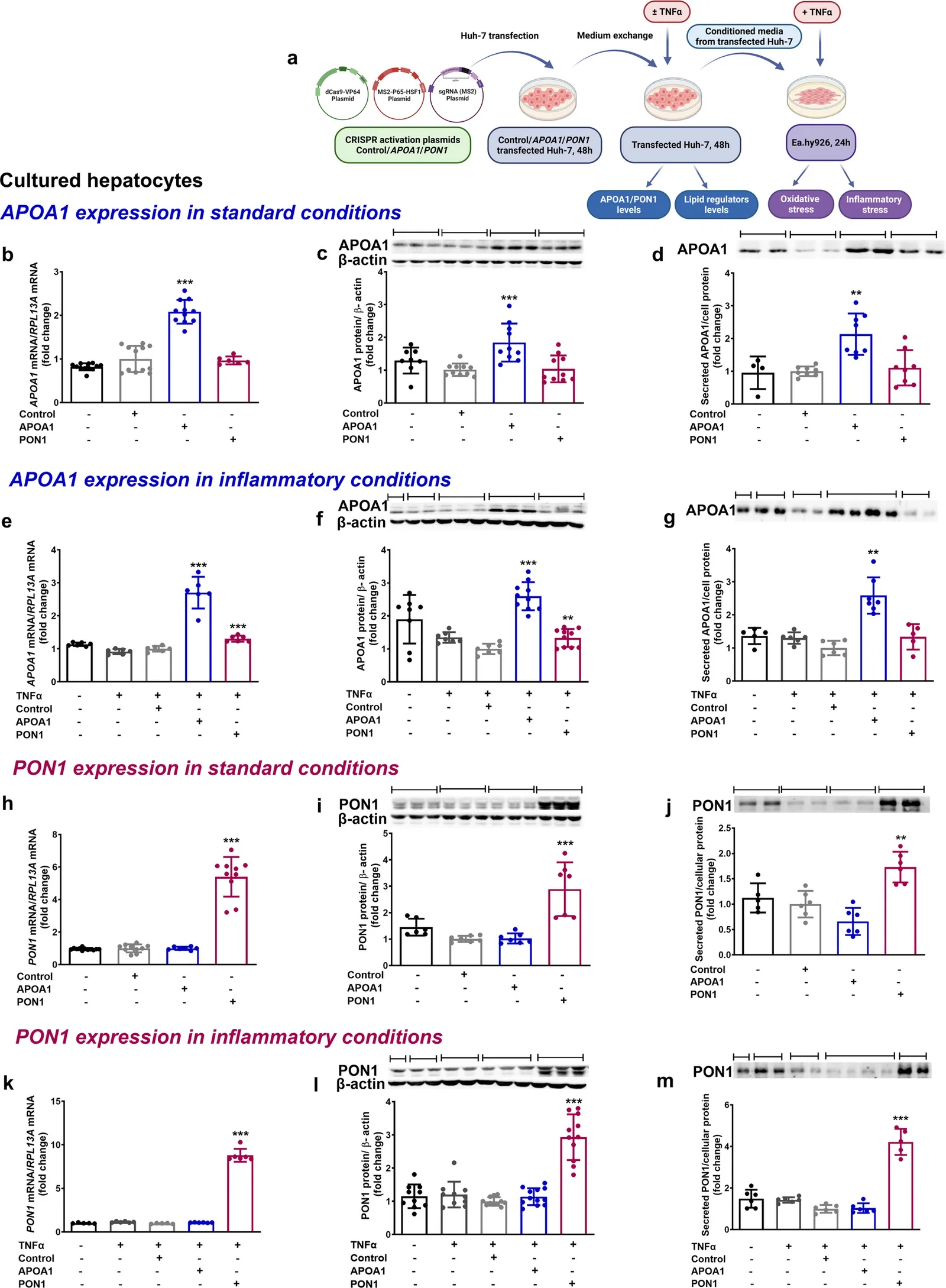
*APOA1* and *PON1* transcriptional activation by CRISPR/dCas9 system in Huh7 cells under standard or pro-inflammatory conditions. Diagram of the in vitro experimental design (**a**) (created with BioRender), *APOA1* mRNA (**b**), intracellular protein (**c**) and secreted protein (**d**) in Huh7 transfected cells in standard conditions or in the presence of tumor necrosis factor α (TNFα) (**e**, **f**, **g**). *PON1* mRNA (**h**), intracellular protein (**i**) and secreted protein (**j**) in Huh7 transfected cells in standard conditions or in the presence of TNFα (**k**, **l**, **m**). Levels of cell protein are represented relative to β-actin, while the levels of secreted proteins in the culture medium are normalized to total cell protein. Results are represented as fold change versus cells transfected with Control plasmids and expressed as mean ± SD of 3–5 independent experiments. **p < 0.01, ***p < 0.001 versus Control

To activate the transcription of endogenous target genes, Huh7 hepatocytes were transfected with the CRISPR/dCas9 construct designed to activate transcription of the *APOA1* and *PON1*, as described under Material and Methods. The experimental design is represented in Fig. 1a. Following transfection, *APOA1* levels were measured by RT-qPCR and Western blotting (WB). Figure 1b shows that *APOA1* gene expression was twofold up-regulated relative to the control plasmid-transfected cells (Control) (*p* < 0.001). In good agreement, Fig. 1c shows an 80% increase in APOA1 protein content in Huh7 cells lysates in comparison with the Control cells (*p* < 0.001). Moreover, Fig. 1d shows that the APOA1 protein secreted in the culture medium was twofold higher versus Control cells (*p* < 0.01). Post-transfection exposure of the Huh7 cells to 30 ng/ml tumor necrosis factor α (TNFα) for 24 h did not alter the transcriptional activation and secretion of APOA1 (Fig. 1e-g).

The Fig. 1h-j show a significant 5.4-fold increase (*p* < 0.001) in *PON1* gene expression in Huh7 cells following transfection with CRISPR/dCas9 plasmids for PON1 activation, compared to that in the Control cells. The activated *PON1* gene expression resulted in a threefold upregulation in the expression of PON1 protein versus Control cells (*p* < 0.001), and secreted PON1 level in the medium showed a 1.73-fold increase over the Control cells (*p* < 0.01). The impact of post-transfection exposure of the cells to TNFα on PON1 expression and secretion is depicted in Fig. 1k-m. The PON1 mRNA level was eightfold higher in comparison with the TNFα-treated Control cells (*p* < 0.001). The protein expression and secreted PON1 levels were also elevated in TNFα-exposed hepatocytes 3- and fourfold, respectively, higher than the Control + TNFα cells (*p* < 0.001 for both parameters). Thus, the results emphasize that the successful transcriptional stimulation of *APOA1* or *PON1* by CRISPR/dCas9 system was not affected by the presence of TNFα.

### Increased expression of APOA1 or PON1 augmented the expression of cholesterol transporters and their regulator sirtuin 1 in Huh7 cells maintained under standard or inflammatory conditions

**Fig. 2:**
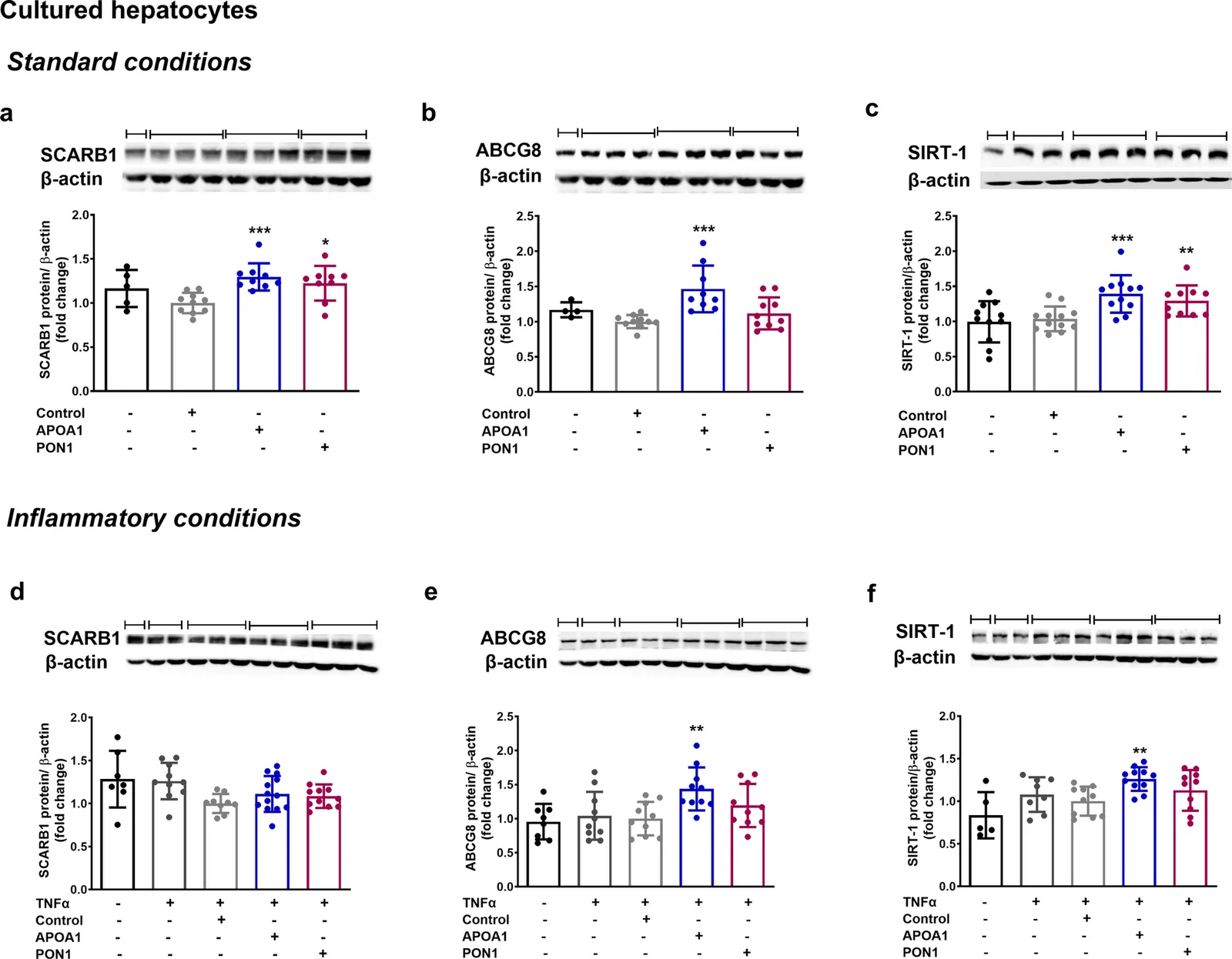
Protein expression of cholesterol transporters and sirtuin 1 in Huh7 cells with upregulated *APOA1* or *PON1* under standard or pro-inflammatory conditions. Protein expression of scavenger receptor class B type 1 (SCARB1) (**a**), ATP-binding cassette sub-family-G-member-8 (ABCG8) (**b**), and sirtuin 1 (SIRT-1) (**c**) in Huh7 transfected cells in standard conditions or under pro-inflammatory conditions (**d**, **e**, **f**) expressed relative to β-actin (blots and their densitometric analysis). Results are represented as fold change compared to cells transfected with Control plasmids and expressed as mean ± SD of 3–5 independent experiments. *p < 0.05, **p < 0.01, ***p < 0.001 versus Control

To answer whether augmented *APOA1* or *PON1* expression could modulate the expression of cholesterol transporters in hepatocytes, we measured the protein expression of the hepatic scavenger receptor class B type 1 (SCARB1), ATP-binding cassette sub-family-G-member-8 (ABCG8) transporter and sirtuin 1 (SIRT-1) in the cells with upregulated *APOA1* or *PON1* in comparison with cells transfected with Control plasmids. The results indicated that the upregulation of *APOA1* was associated with moderate, yet significant, increase of both SCARB1 (30%, *p* < 0.001) and ABCG8 (45%, *p* < 0.01) protein expression, whereas PON1 upregulation was less effective and was associated with 20% increase of SCARB1 (*p* < 0.05), in comparison with Control cells (Fig. 2a, b). SIRT-1 protein expression was increased in Huh7 cells presenting upregulated *APOA1* or *PON1* by 37% (*p* < 0.001) and 28%, respectively (*p* < 0.01) (Fig. 2c).

The SCARB1 protein level in upregulated *APOA1* cells remained comparable to the levels measured in the Control cells exposed to TNFα (Fig. 2d). Interestingly, the expression levels of ABCG8 and SIRT-1 were increased by 43% and 26%, respectively (*p* < 0.01 for both) in TNFα-treated Huh7 cells presenting upregulated *APOA1*, in comparison with TNFα-treated Control cells (Fig. 2e, f). Noteworthy, the combination of *PON1* upregulation with TNFα treatment did not stimulate significantly the expression of SCARB1, ABCG8 or SIRT-1 (Fig. 2d-f). These results confirm that upregulated *APOA1* or *PON1* have positive effects on the gene and protein expression of lipid transporters in transfected hepatocytes.

### Conditioned medium from Huh7 cells with upregulated APOA1 ameliorated oxidative and inflammatory status of cultured vascular EC exposed to TNFα

**Fig. 3:**
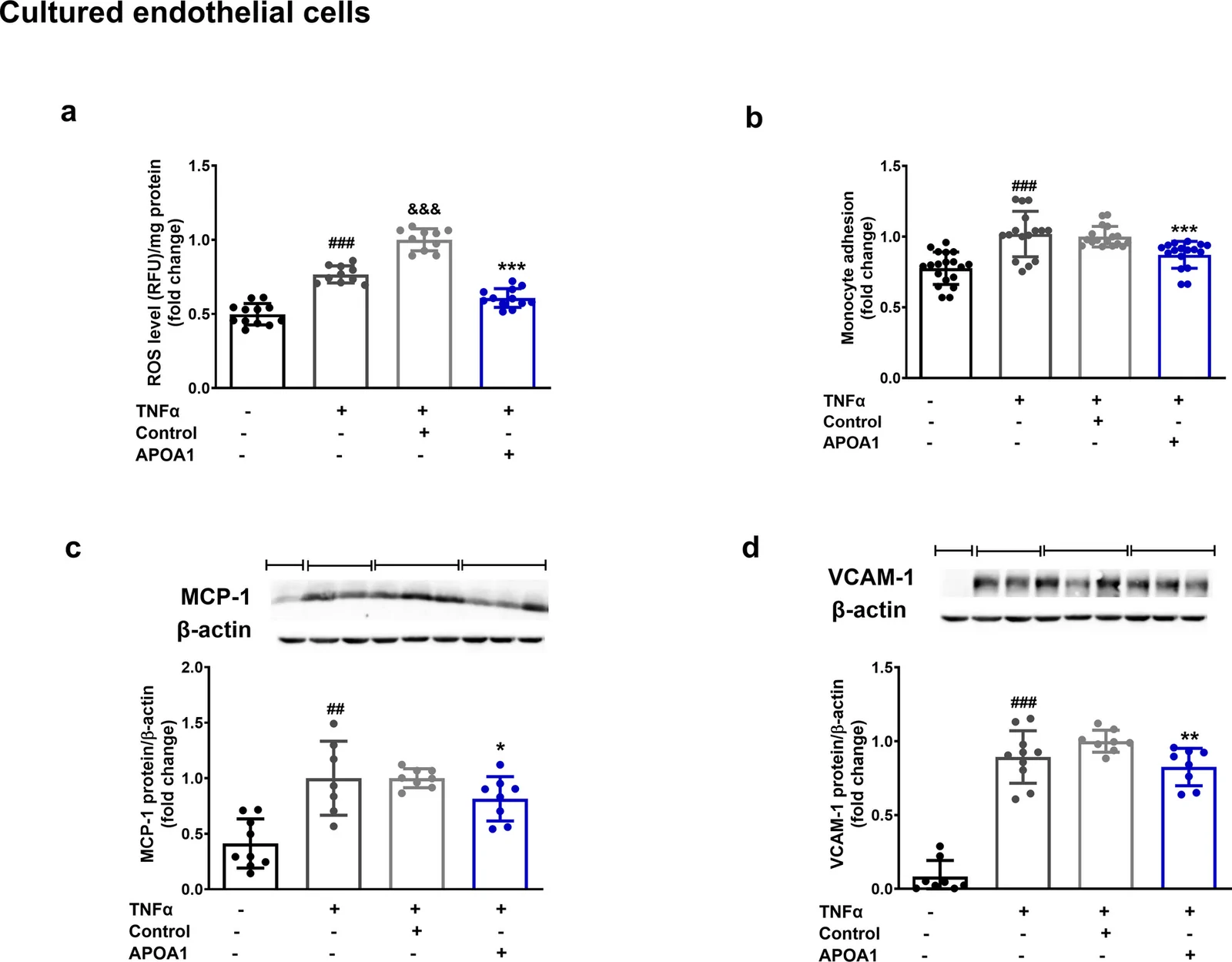
Oxidative and inflammatory stress in TNFα-activated endothelial cells incubated with conditioned media from Huh7 cells with upregulated *APOA1*. Cellular reactive oxygen species (ROS) production expressed relative to cellular protein (**a**), THP-1 monocytes adhesion to activated EC (**b**), and protein expression of monocyte chemoattractant protein-1 (MCP-1) (**c**) and vascular cell adhesion molecule-1 (VCAM-1) (**d**) expressed relative to β-actin (blots and their densitometric analysis). Results are represented as fold change compared to cells transfected with Control plasmids and presented as mean ± SD of 3–5 independent experiments. *p < 0.05, **p < 0.01, ***p < 0.001 versus Control; ^##^*p* < 0.01, ^###^*p* < 0.001 versus un-transfected cells not exposed to TNFα; ^&&&^p < 0.001 versus un-transfected cells exposed to TNFα

To evaluate the protective effects of secreted APOA1, oxidative and inflammatory stress were assessed in TNFα-treated EC exposed for 24 h to conditioned medium (CM) that was prepared from Huh7 cells with upregulated *APOA1*. Figure 3a shows that CM from Huh7 with upregulated *APOA1* decreased TNFα-induced intracellular reactive oxygen species (ROS) by 40% as compared to CM from Control hepatocytes (*p* < 0.001). It is also important to note that the CM from these Control cells increased ROS production above the basal levels that were determined in cells that were not treated with TNFα or treated with TNFα but unexposed to CM.

The Fig. 3b-d summarizes the impact of the APOA1-enriched CM on the adhesion of THP-1 monocytes and monocyte chemoattractant protein-1 (MCP-1) and vascular cell adhesion molecule-1 (VCAM-1) protein expression levels in EC, under similar experimental conditions as described under Fig. 1a. The results showed a slight decrease of monocyte adhesion to TNFα-treated EC (13% versus Control, *p* < 0.001) (Fig. 3b). In good agreement, the protein expression levels of MCP-1 and VCAM-1 were significantly decreased by the CM enriched in APOA1 (19% for MCP-1, *p* < 0.05; 18% for VCAM-1, *p* < 0.01) (Fig. 3c, d). The CM from un-transfected Huh7 cells induced a 50% increase of ROS production in EC, and has no effect on monocyte adhesion (Fig. S1). These data indicate that APOA1-enriched CM exerts antioxidant and anti-inflammatory actions in activated EC.

### ApoE^−/−^mice treated with *Apoa1*- or *Pon1*- CRISPR/dCas9 system exhibited elevated levels of APOA1 or PON1 in the liver and sera; alleviation of oxidative and inflammatory stress

**Fig. 4:**
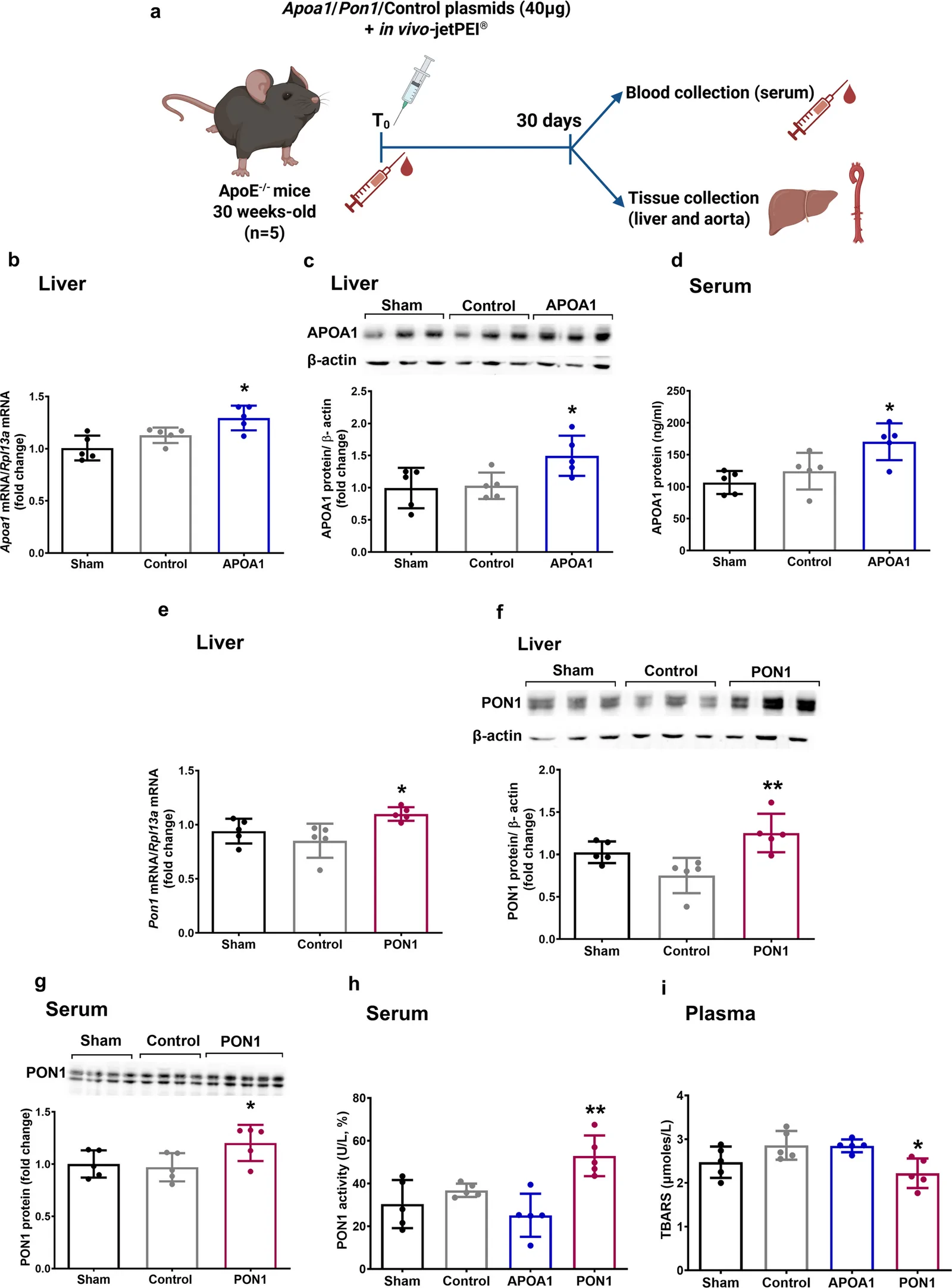
Gene expression and protein levels of *Apoa1* and *Pon1* in the liver and sera of apoE^−/−^ mice injected with CRISPR/dCas9 plasmids for their transcriptional activation. Diagram of the in vivo experimental design (**a**) (created with BioRender), liver *Apoa1* mRNA (**b**), liver APOA1 protein (**c**), APOA1 level in sera (**d**), liver *Pon1* mRNA (**e**), liver PON1 protein (**f**), PON1 protein levels in sera (**g**), PON1 enzymatic activity in sera (**h**), and thiobarbituric acid reactive substances (TBARS) levels in the plasma of the mice (**i**). The data in b, c, e, f and g are expressed as fold change compared to Control group. All data are presented as mean ± SD (*n* = 5); *p < 0.05, ***p < 0.001 versus mice injected with Control plasmids

Recent studies indicate that the liver is one of the organs targeted by the CRISPR/dCas9 – in vivo-jetPEI system following i.v. injection [20]. It is well established that the liver is the primary source of APOA1 and PON1 [2]. The in vivo experimental design is represented in Fig. 4a, and the results in Fig. 4b, c, e, f show that the levels of *Apoa1* and *Pon1* were significantly increased in mice injected with the relevant activation plasmid systems compared to Control group. Specifically, 30%, and 25% (*p* < 0.05 for both) increase for hepatic *Apoa1* and *Pon1* mRNA, respectively; 40%, and 55% (*p* < 0.05 for APOA1 and *p* < 0.001 for PON1) for APOA1 and PON1 protein, respectively. Concomitantly, APOA1 and PON1 protein levels were significantly increased in the sera of mice injected with activation plasmids for *Apoa1* or *Pon1* respectively, compared to Control group (37%, *p* < 0.05 for APOA1, Fig. 4d and 20%, *p* < 0.05, for PON1, Fig. 4g).

The serum level of TNFα was significantly decreased, while the interlekin-10 (IL-10) level was increased in mice with upregulated *Apoa1* compared to Control (19%, *p* < 0.05 for TNFα, 140%, *p* < 0.001 for IL-10), indicating a lessened inflammatory stress (Table 1). Importantly, 30 days after the CRISPR/dCas9 plasmids injection, a significant increase of PON1 activity was still prominent in the sera of mice from the PON1 group, compared to Control mice (40%, *p* < 0.05) (Fig. 4h). Plasma thiobarbituric acid-reactive species (TBARS) level and serum TNFα concentration were significantly decreased, while the serum level of anti-inflammatory IL-10 was enhanced in mice with upregulated *Pon1* compared to Control mice (22%, *p* < 0.05 for TBARS, 18%, *p* < 0.05 for TNFα, 55%, *p* < 0.05 for IL-10) (Fig. 4i, Table 1), suggesting an increase in the antioxidant capacity and a decrease of inflammatory stress in the blood of PON1 mice.

**Table 1:** Biochemical parameters in the sera and liver of apoE^−/−^ mice injected with CRISPR/dCas9 plasmids for *Apoa1* or *Pon1* transcriptional activation

Parameter	Sham (*n* = 5)	Control (*n* = 5)	APOA1 (*n* = 5)	PON1 (*n* = 5)
Serum				
TNFα (pg/mL)	173.22 ± 4.98	182.33 ± 23.57	147.88 ± 10.82*	150.48 ± 16.28*
IL-10 (ng/mL)	1.19 ± 0.31	1.02 ± 0.42	2.51 ± 0.21***	1.58 ± 0.37*
Creatinine (µg/mL)	439.02 ± 61.38	414.63 ± 116.88	421.14 ± 78.06	429.27 ± 68.36
AST (U/L)	53.71 ± 4.7	37.80 ± 8.80	31.34 ± 6.56	37.66 ± 6.38
ALT (U/L)	32.39 ± 5.53	35.78 ± 3.50	26.89 ± 6.87^*^	41.12 ± 5.18
Total Cholesterol (mg/dL)	351.39 ± 35.08	305.67 ± 60.87	389.48 ± 29.42*	297.58 ± 58.47
HDL-Cholesterol (mg/dL)	29.14 ± 5.64	32.10 ± 3.03	38.89 ± 4.8*	31.48 ± 5.00
Liver homogenate				
Total cholesterol (µg/mg protein)	26.12 ± 3.37	26.08 ± 2.53	29.58 ± 2.47^*^	27.44 ± 1.72

All data are presented as mean ± SD; *p < 0.05, ***p < 0.001 versus mice injected with Control plasmids

To explore the impact of CRISPR/dCas9 plasmids + in vivo*-*jetPEI® on the renal and hepatic functions of apoE^−/−^ mice, we assessed the serum levels of creatinine, aspartate aminotransferase (AST) and alanine aminotransferase (ALT) at 30 days after the injection. Table 1 shows no significant increase of these parameters in sera, suggesting that plasmid administration did not induce hepatic and renal toxicity. Additionally, to evaluate whether jetPEI/plasmid cocktail induces innate immune responses, the gene expression of *Il6* and *Ifnb1* and IL-6 protein levels were estimated in the liver and sera of apoE^−/−^ mice. No increase of all these parameters was detected (Fig. S2a-d). These results show that the transcription of endogenous *Apoa1* and *Pon1* can be successfully activated in the liver of apoE^−/−^ mice by using the CRISPR/dCas9 system without inducing renal and hepatic dysfunction or immunogenicity, and the elevated levels of their proteins in the circulation are associated with alleviated oxidative and inflammatory stress.

### Serum HDL-cholesterol and total cholesterol were elevated in apoE^−/−^mice with upregulated *Apoa1*; distribution of APOA1 between IDL/LDL, HDL, and the lipid-free form

To evaluate the effect of upregulated *Apoa1* on the cholesterol level in serum, we assessed the total cholesterol (TC) and HDL-C concentration. Table 1 shows that the TC and HDL-C levels were significantly increased in the sera of mice treated with *Apoa1* activation plasmids in comparison with the Control group (27% and 21%, respectively, *p* < 0.05). However, upregulated *Pon1* in the mice did not induce changes in the TC and HDL-C in sera.

**Fig. 5:**
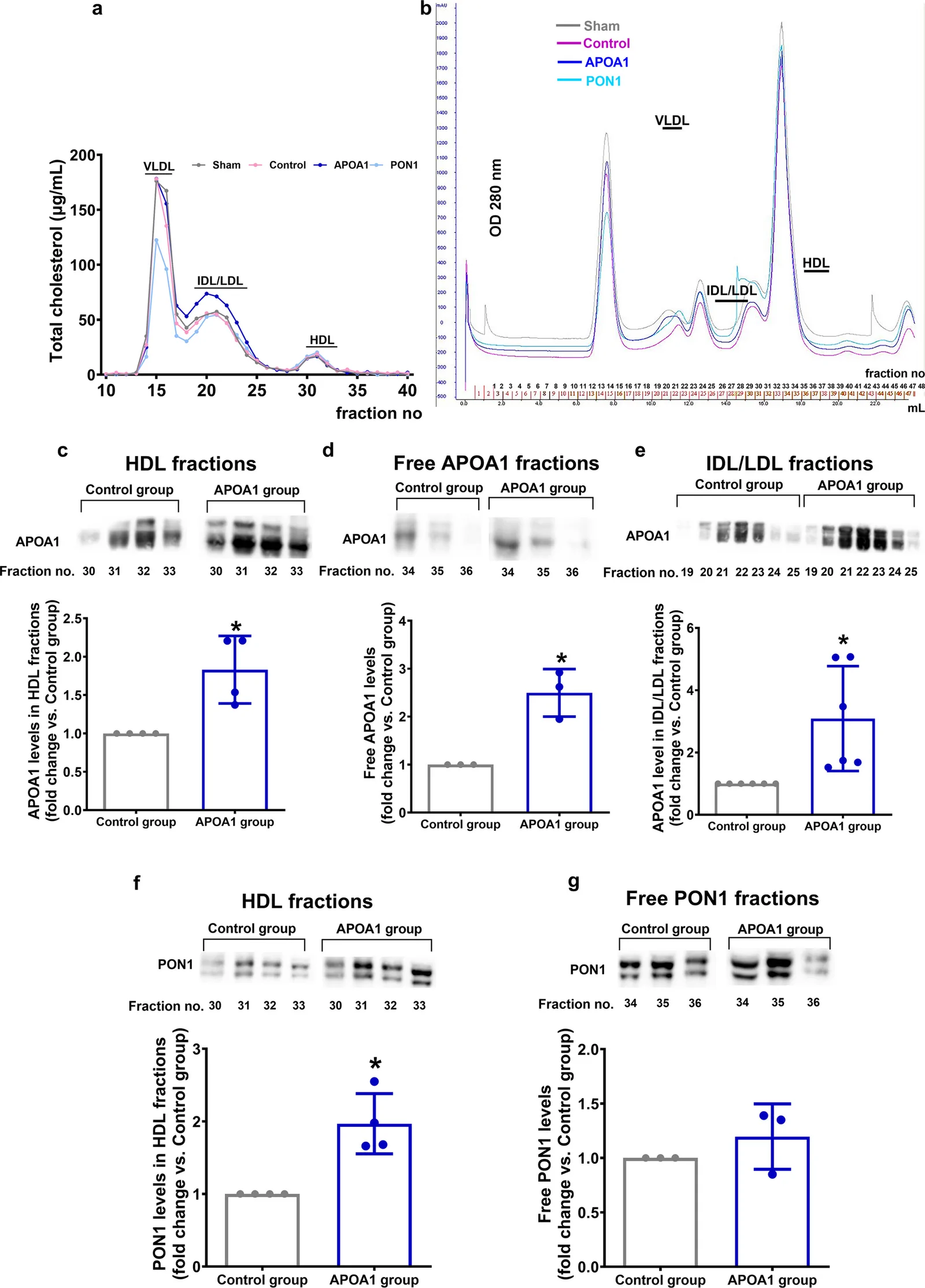
The levels of total cholesterol, APOA1 and PON1 in lipoproteins separated by FPLC from plasma of apoE^−/−^ mice injected with CRISPR/dCas9 plasmids for *Apoa1* activation. Lipoprotein FPLC profiles analyzed as total cholesterol (**a**), protein profile measured at 280 nm (**b**), blots and their densitometric analysis representing the levels of HDL-bound APOA1 (fractions no. 30–33) (**c**), lipid-free APOA1 (fractions no. 34–36) (**d**), APOA1 in intermediate-density lipoproteins/low-density lipoproteins (IDL/LDL) (fractions no. 19–25) (**e**), HDL-bound PON1 (fractions no. 30–33) (**f**), and free PON1 (fractions no. 34–36) (**g**). All data are expressed as fold change relative to the equivalent fraction of the Control group and presented as mean ± SD (technical replicates of 3–7 fractions from pool of 5 plasma); *p < 0.05 versus mice injected with Control plasmids

To investigate the distribution of increased circulating APOA1 between lipoproteins, we separated by fast protein liquid chromatography (FPLC) the fractions from pooled plasma of each experimental group. The Fig. 5 represents the FPLC profile indicating no shift of the lipoprotein fractions among groups, determined as cholesterol (a) or protein level measured at 280 nm (b). A 30% increase of the cholesterol level in the intermediate-/low-density lipoprotein (IDL/LDL) fractions was measured in the case of APOA1 mice compared to Control group (Fig. 5a). A significant increase of APOA1 levels was detected in HDL (90% for fractions 30–33, *p* < 0.05) (Fig. 5c), but also as lipid-free form (130% for fractions 34–36, *p* < 0.05) versus Control (Fig. 5d). Very interesting results were obtained for IDL/LDL fractions, in which a surprisingly increased content of APOA1 was detected (200% for fractions 19–25, *p* < 0.05), compared to the corresponding fractions from the Control group (Fig. 5e). In animals with upregulated APOA1, the level of PON1 associated with HDL was elevated by 100% compared to the equivalent fractions in the Control group, suggesting that the above-mentioned increase of APOA1 level in HDL determined the augmented level of HDL-associated PON1 (Fig. 5f). No significant changes were detected for the level of free PON1 in APOA1 mice (Fig. 5g) or in the content of HDL-bound PON1 and free PON1 in mice injected with activation plasmids for *Pon1* (Fig. S3). These results indicate that the increased free APOA1 is a source for enhanced APOA1 content of HDL, which in turn leads to increased attachment of PON1 to HDL. Unexpectedly, the upregulation of *Apoa1* generated an enrichment of LDL fraction with APOA1.

### Upregulation of *Apoa1* in the liver of apoE^−/−^ mice modulates the hepatic SCARB1, ABCG8 and CYP7A1, and the bile and feces cholesterol content

**Fig. 6:**
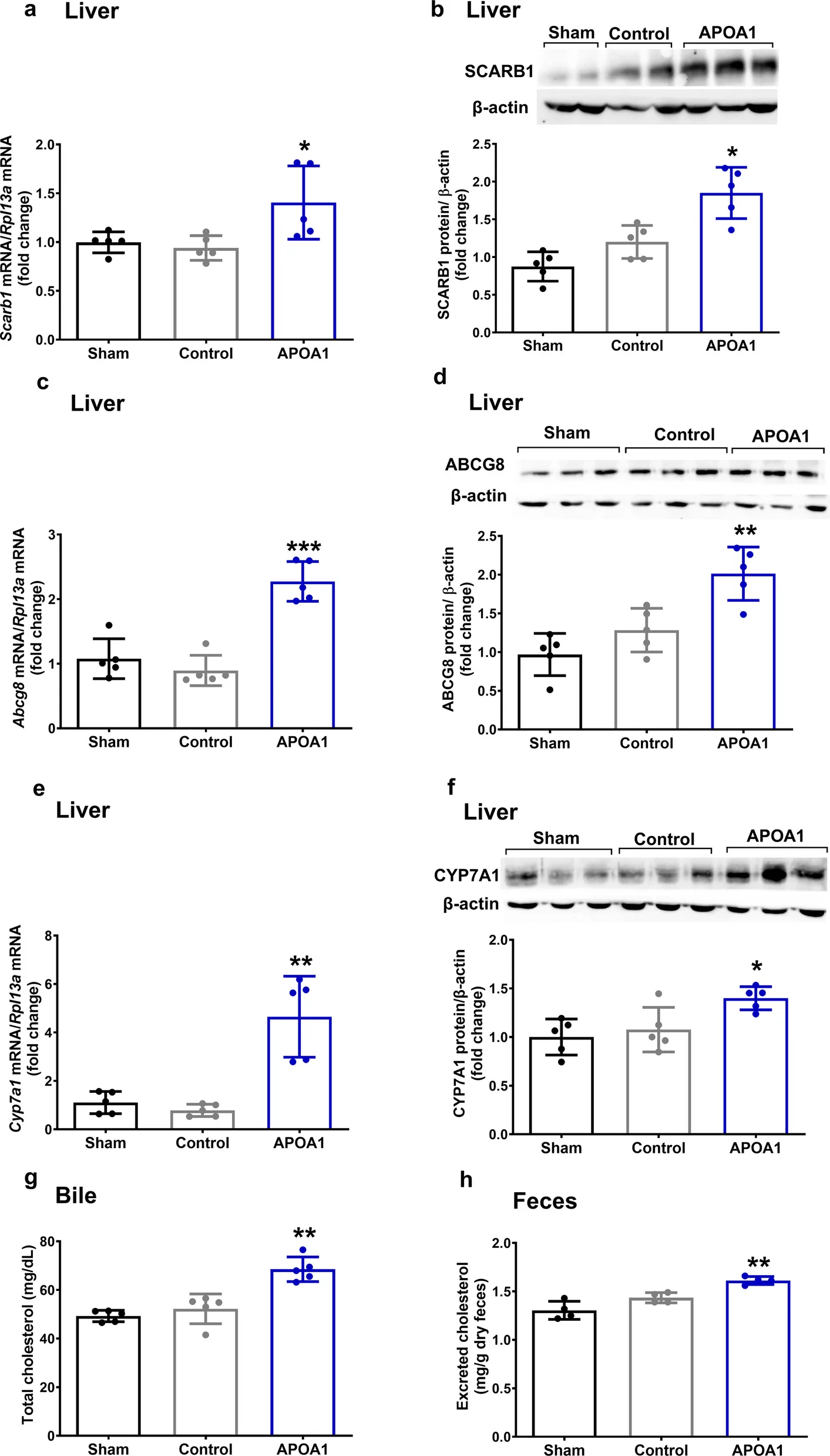
Gene and protein expression of cholesterol transporters SCARB1 and ABCG8, and CYP7A1 in the liver of apoE^−/−^ mice injected with *Apoa1* CRISPR/dCas9 plasmids. *Scarb1* mRNA (**a**) and protein (**b**) levels, *Abcg8* mRNA (**c**) and protein (**d**) levels, *Cyp7a1* mRNA (**e**) and protein (**f**) levels, total cholesterol content of the bile (**g**) and feces (**h**). The data in a-f are expressed as fold change compared to Control group. All data are presented as mean ± SD (*n* = 5); *p < 0.05, **p < 0.01, ***p < 0.001 versus mice injected with Control plasmids

The impact of the upregulated *Apoa1* and *Pon1* on the hepatic cholesterol transport in ApoE^−/−^ mice was assessed by measuring the levels of SCARB1, ABCG8 and cholesterol 7 alpha-hydroxylase (CYP7A1). TC was measured in the liver, bile and feces of the mice. The mRNA and protein levels of SCARB1, ABCG8, and CYP7A1 were increased in the mice from APOA1 group as follows: 48% and 55% for SCARB1, respectively, 100% and 30% for ABCG8, respectively, and 600% and 30% for CYP7A1 (Fig. 6a-f). The TC levels measured in the liver, bile and feces of APOA1 mice were increased by 14%, 30% and 12%, respectively (*p* < 0.05) in comparison with the Control group (Table 1 and Fig. 6g, h). These data point to an enhancement of TC excretion through bile. In contrast to these APOA1-asssociated effects, there was no evidence for similar effects of the upregulated *Pon1* in the mice, as the protein levels of SCARB1, ABCG8 or CYP7A1 did not vary.

### Upregulation of *Apoa1* or *Pon1* in apoE^−/−^ mice markedly reduced the lipid deposits area in the thoracic aorta and the aortic root

**Fig. 7:**
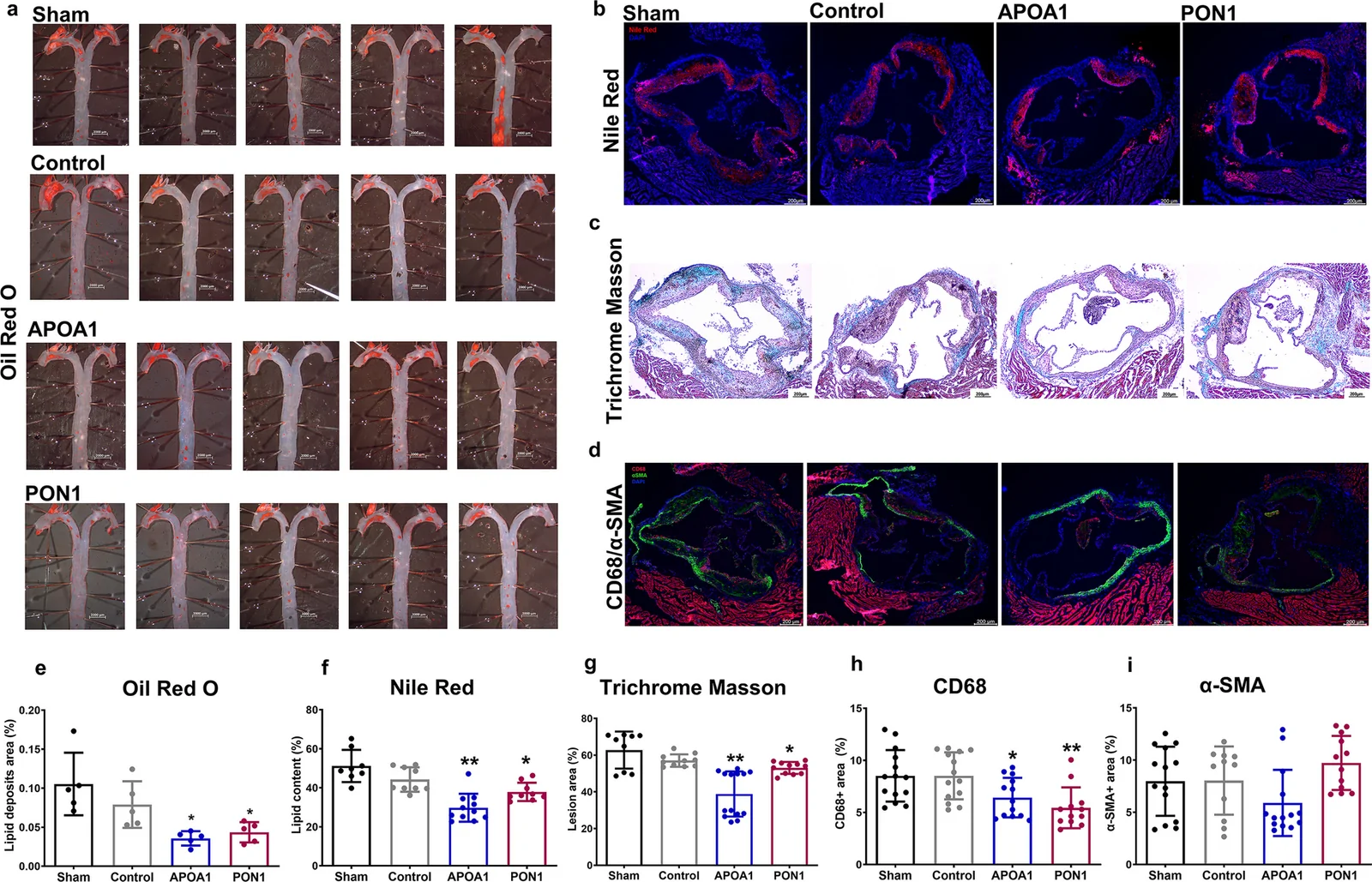
Lipid deposits area in the lesions located in the aorta and aortic root of apoE^−/−^ mice injected with *Apoa1* or *Pon1* CRISPR/dCas9 plasmids. Light microscopy images of the thoracic aorta stained with Oil Red O, scale bar: 2000 μm (**a**), lipid content of the aortic root lesions evidenced by Nile Red staining (**b**), sections of the aortic root stained with Trichrome Masson (**c**), specimens of the aortic root immunostained for CD68 (red fluorescence) and α-SMA (green fluorescence) (**d**), diagram presenting the ratio between the area of Oil Red O stained lipid deposits and the total area of the thoracic aorta, data presented as mean ± SD (*n* = 5) (**e**), diagram presenting the percentage of Nile Red-positive area to the total aortic wall area from 3–5 consecutive sections per animal (*n* = 3) (**f**), diagram presenting the size of the aortic root lesions represented as the percentage relative to the total aortic wall area from 3–5 consecutive sections per animal (*n* = 3) (**g**), diagram presenting the size of the CD68 (**h**) and α-SMA (**i**) positive area represented as percentage of the total area of lesions from 4 consecutive sections per animal (*n* = 3). All data from the aortic root images are presented as mean ± SD and the scale bar is 200 μm; *p < 0.05 and **p < 0.01 versus mice injected with Control plasmids

To evaluate the effect of the upregulated *Apoa1* or *Pon1* on the lesions area and their lipid deposits, the thoracic aortae were stained with Oil Red O. The images in Fig. 7a and the analysis showed a 50% reduction (*p* < 0.05) of the areas of the lipid deposits in the aortae of the APOA1 mice, and a 39% reduction (*p* < 0.05) in the PON1 mice in comparison with the Control animals (Fig. 7d). The neutral lipid content of the lesions in the aortic root decreased by 30% (*p* < 0.001) in APOA1 mice, and by 16% (*p* < 0.05) in PON1 mice, as was evidenced by Nile Red staining (Fig. 7b, e). The area of the lesions located in the aortic root was diminished by 30% (*p* < 0.001) in APOA1 mice, and by 10% (*p* < 0.05) in PON1 mice (Fig. 7c, f). To evaluate plaque composition, the aortic roots cryosections were immunostained for CD68 as marker for macrophage infiltration, and for α-smooth muscle actin (α-SMA) as a marker of vascular smooth muscle cells. The CD68 + area was significantly reduced both in APOA1 and PON1 groups (by 25%, *p* < 0.05, and 35%, *p* < 0.01, respectively), while the α-SMA + area was not significantly modified compared to Control group (Fig. 7d, h, i). These data indicate that the anti-atherosclerotic effect of upregulated *Apoa1/Pon1* consists of the reduction of lesions’ area and their lipid content, paralleled by a reduced inflammatory process within the lesions.

## Discussion

It has long been suggested that improving HDL quality and quantity may become an essential therapeutic strategy to alleviate the induction and progression of atherosclerosis [2]. Many attempts of using HDL mimetics, weekly administrated, to treat CVD have failed to demonstrate their therapeutic potential due to adverse events or deficiency in inducing regression of atherosclerotic lesions [21, 22]. CRISPR/Cas9 was used to down-regulate various regulatory genes, such as proprotein convertase subtilisin/kexin type 9, LDL receptor, or angiopoietin-like 3, with some promising results to reduce the cardiovascular burden, especially in familial hypercholesterolemia [23].

The main aim of the study, based on our previous discoveries about dysfunctional HDL in CVD patients [6, 8], was to improve the protein composition of HDL and to validate its anti-atherosclerotic function in apoE^−/−^ mice model of spontaneous atherosclerosis. The present study introduces an entirely different approach: to use CRISPR/dCas9 system to augment the athero-protective effects of HDL by increasing the expression of endogenous APOA1 and PON1. Thus, we employed CRISPR/dCas9 technology to increase APOA1 and PON1 in hepatocytes in culture and in apoE^−/−^ mice.

The main findings of the present study are that the transcription of endogenous APOA1 and PON1 can be successfully activated in cultured hepatocytes and in apoE^−/−^ mice using the CRISPR/dCas9 system. The mechanisms mediating the beneficial effects of upregulated APOA1 and PON1 are: (i) the conditioned medium of APOA1-transfected hepatocytes protects TNFα-activated EC by reducing ROS levels, MCP-1 and VCAM-1 protein expression, and monocyte adhesion; (ii) increased APOA1 in HDL leads to additional PON1 recruitment and contributes to increased HDL atheroprotection; (iii) increased HDL-C levels stimulates the pathway for liver cholesterol removal through bile via upregulation of hepatic *Scarb1*, *Abcg8* and *Cyp7a1*; (iv) increased LDL-associated APOA1 may impede LDL retention in the arterial wall; (v) increased PON1 antioxidant activity, leads to decreased plasma oxidative and inflammatory stress (reduced TBARS and TNFα, increased IL-10). Most important, the liver secreted APOA1 unattached to or associated to lipoproteins may impede the development of lipid deposits in the aorta by exerting anti-oxidant and anti-inflammatory actions, reducing LDL atherogenicity and augmenting the liver cholesterol excretion. Moreover, the functional validation of the above mechanisms is the 50% for APOA1 and 40% for PON1 endpoint reduction of the lipid deposits area in the thoracic aortae of the treated mice, supporting well the aim of the study.

Over the last decade, the improvement of HDL quantity and quality was proposed as a therapeutic strategy to arrest the evolution of the atherosclerotic process. While initial studies showed that HDL infusion reduced aortic lesions in cholesterol-fed animals, studies in humans proved that HDL mimetics failed to protect patients post-myocardial infarction [14, 15]. There are some presumed reasons for the failure of these studies including the variability of HDL mimetics preparation, the short lifespan of HDL infusions in the circulation and possibly the oxidative alteration of APOA1 typical for pathological conditions in CVD [24, 25].

APOA1 mediates RCT from the peripheral tissues to the liver through interactions with hepatic SCARB1 receptors. In addition, it has been shown to have anti-inflammatory and anti-oxidant properties [2, 7, 9, 26]. PON1 secreted in the circulation associates with HDL [2], and in the context of CVD, it was shown that PON1 inhibits the lipid oxidation in HDL and LDL, and favors the cholesterol efflux from macrophages [2, 13, 27]. Interesting, it was shown that PON1 activity is not entirely dependent on its association with HDL, published data showing that cellular membranes can acquire PON1 which remains active and protects the cells from oxidative stress damages [13, 28].

The present data shows that the transcriptional activation of APOA1 or PON1 by CRISPR/dCas9 system successfully and effectively increased the expression of these two proteins and doubled their secretion in the culture medium of hepatocytes. We demonstrate here that the upregulation of *APOA1* and *PON1* persists in hepatocytes even under inflammatory conditions, and the transcription, translation and secretion of these two proteins remain elevated. More important, the study shows here for the first time, by using the CRISPR/dCas9 system, that endogenous APOA1 and PON1 protein levels can be increased in the liver and sera of mature apoE^−/−^ mice, a well-known model of atherosclerosis. The upregulation of *Apoa1* and *Pon1* was accomplished by a single dose of the specific activating plasmids, without cytotoxic or immunogenic effects, and their augmented levels remained elevated one month after the injection. Very interesting, the elevated APOA1 in serum was distributed between IDL/LDL, HDL and the lipid-free form of the protein. The lipid-free APOA1 may function as a reservoir of protective protein that exerts its role by multiple pathways. Thus, the lipid-free form may facilitate the exchange of APOA1 among various populations of HDL and mediate RCT [29]. APOA1 and HDL can bind to SCARB1 with high affinity and continuously compete with LDL to SCARB1 binding [30]. Therefore, APOA1/HDL may limit SCARB1-mediated LDL trancytosis, thus helping to slow the development of atherosclerosis. Recently, it was reported that lipid-free APOA1, or weakly attached to HDL particles, can transfer to LDL particles, making the latter less atherogenic by impeding their binding to proteoglycans and consequently, reducing LDL retention in the arterial wall [10]. The FPLC data prove that the LDL fraction was enriched in APOA1, most likely due to the increased lipid free APOA1 or the APOA1 that enriches HDL particles. These findings shed new light on LDL interactions and support new mechanisms for APOA1 anti-atherogenic action. It is worth noting that an increase in APOA1 level in HDL allows an addition of PON1 on the HDL particles that certainly makes them more athero-protective as was previously shown [6, 8]. The increased level of serum APOA1 was associated with a moderate increase of HDL-C level and a reduction of inflammatoy stress measured as decreased TNFα and increased IL-10 in APOA1 mice.

In the sera of mice injected with CRISPR/dCas9 plasmids for *Pon1* activation, the protein levels and activity of the target enzyme were increased, conferring a more powerful antioxidant protection in good agreement with the decrease of the oxidative stress measured as plasma TBARS levels. As already known, the oxidative and inflammatory stress are closely related, and the upregulation of *Pon1* was associated with a reduction of the inflammatoy stress (decreased TNFα, increased IL-10) in apoE^−/−^ mice, suggesting that PON1 anti-oxidant effect is accompanied by anti-inflammatory outcome, leading to diminished aortic lipid deposits. It is known that the attachment of PON1 to HDL is dependent on the level and quality of APOA1 present in HDL particles [31]. The level of PON1 in HDL was not increased in mice from PON1 group, probably due to the absence of a consistent increased APOA1 that would favor its attachment to HDL, as was in mice from APOA1 group. It is noteworthy that PON1 protein levels remained elevated in the liver one month after the injection, this result suggesting that PON1 may also act attached to the cells.

Exploring the cholesterol homeostasis in the liver, we analyzed hepatic SCARB1, ABCG8 and CYP7A1. SCARB1 is a multifunctional receptor, involved in the promotion of RCT, mediating the selective uptake of the cholesterol esters from HDL in the liver and the biliary cholesterol secretion [32]. SCARB1 deficiency has been associated with accelerated atherosclerosis in mice and with a reduced secretion of cholesterol in the bile [32, 33]. Together with SCARB1, the heterodimer ABCG5/8 is involved in the excretion of hepatic cholesterol into the bile [34–36].

In cultured hepatocytes, the upregulation of *APOA1* determines the increase of both SCARB1 and ABCG8 protein expression, in good agreement with the results obtained previously by our group in cultured enterocytes [37]. To identify the liable mechanisms, we assessed the expression of SIRT-1, known as indirect positive regulator for SCARB1 and ABCG8 in hepatocytes through liver X receptor (LXR) activation [32, 38, 39]. It was shown before that SIRT-1 positively regulates the expression of LXR by mediating its deacetylation at a lysine residue [40]. In turn, LXR is known to activate the promoter of SCARB1 [41] and to directly stimulate the transcription of ABCG8 in hepatocytes [42]. The observed increase of SIRT-1 protein expression by the upregulated *APOA1* could explain the activation of *SCARB1* and *ABCG8* detected in the cultured hepatocytes. The results evidenced in apoE^−/−^ mice that the upregulated *Apoa1* increases the protein expression of SCARB1 and ABCG8 which is associated with an increase of TC levels in the bile and feces, probably due to their concerted action. Another pathway for the elimination of the surplus of cholesterol from the liver is through CYP7A1, an enzyme that transforms liver cholesterol in bile acids necessary for the emulsification of dietary fats [43]. The in vivo results demonstrate an upregulation of *Cyp7a1* levels in the liver of APOA1 mice, suggesting a complementary process for the cholesterol clearance from the liver into the bile.

The in vitro part of the study provides a mechanistic insight into the athero-protective effects that were observed in the animal model. Of particular interest are the findings of a positive crosstalk between hepatocytes and vascular EC. Under inflammatory conditions, EC express increased levels of cytokines and surface adhesion molecules that favor the attachment and diapedesis of immune cells from blood to the subendothelial space, inducing and exacerbating the atherosclerotic process [1]. The here presented results evidence for the first time the impact of CRISPR/dCas9-upregulated *APOA1* in hepatocytes, demonstrating that the conditioned medium containing elevated levels of APOA1 induces the decrease of ROS levels, VCAM-1 and MCP-1 expression and monocyte adhesion in TNFα-activated EC.

We here validate the functionality of endogenously upregulated *Apoa1* and *Pon1* in a mouse model of spontaneous atherosclerosis, resulting in an endpoint reduction of the lipid deposits, with a decreased inflammatory response in the aorta and aortic root of apoE^−/−^ mice.

Our study has limitations regarding the small sample size of animal groups, pooled FPLC samples, limited aortic plaque characterization, the duration of the experiment, and the inferred transcriptional activation. Future research should explore whether the upregulation of APOA1 or PON1 by the CRISPR/dCas9 system can be sustained longer in vivo, potentially through repeated injections.

## Conclusions

The successful upregulation of *Apoa1* and *Pon1* using the CRISPR/dCas9 system determines the increase of these proteins levels in the liver and blood of apoE^−/−^ mice with beneficial effects, such as reduction of the oxidative and inflammatory stress, and lipid deposits in the aorta. These results support future attempts for the use of CRISPR/dCas9 transcriptional activation of endogenous genes to treat atherosclerosis. Further studies are needed for mechanistic deciphering on long-term effects.

## Materials and methods

### Reagents

The sources of general reagents are provided in the Supplemental Information. Human *APOA1*, human *PON1*, mouse *Apoa1*, mouse *Pon1* and Control CRISPR/dCas9 activation plasmids, transfection medium, and UltraCruz Transfection Reagent used are licensed products of Santa Cruz Biotechnology, Inc., CA, USA and facilitate the recognition and transcriptional activation of specific genes by employing the D10A and N863A deactivated Cas9 (dCas9) nuclease that is fused to a VP64 activation domain. The system is combined with a target-specific sgRNA engineered to bind the MS2-P65-HSF1 fusion protein. All these components constitute a synergistic activation mediator (SAM) transcription activation system for each target protein.

### Cell cultures experiments

Hepatocytes from human hepatocarcinoma (Huh7 transformed cell line, Cell Lines Service GmbH, Germany), mycoplasma free, were grown according to manufacturers’ instructions. The short tandem repeat (STR) analysis of these cells evidenced a 94.5%–98.2% genetic match with the CLS reference.

To stimulate *APOA1* or *PON1* transcription, Huh7 cells (70%–80% confluency) were transfected with *APOA1* or *PON1* activation plasmids (1 µg DNA plasmid/mL +2.5µL/mL transfection reagent) as indicated by the manufacturer. Subsequently, the medium was changed to serum-free RPMI for the last 24 h to collect the CM enriched in APOA1 or PON1.

To assess the level of upregulated proteins in transfected hepatocytes exposed to inflammatory stress, at 72 h after the transfection, Huh7 cells were exposed to 30 ng/ml TNFα in serum-free RPMI for 24 h, then were lysed to perform RT-qPCR and WB analysis. The supernatant from transfected Huh7 was centrifuged at 300xg for 5 min, and the obtained CM was stored at −80 °C for further analysis or used in EC functional assays. Untransfected Huh7 cells or Huh7 cells transfected with control CRISPR/dCas9 activation plasmids (Control) were used as references.

Human umbilical vein EC (EA.hy926 transformed cell line, ATCC, Manassas, VA, USA), mycoplasma free, were grown according to manufacturers’ recommendations. The STR analysis of these cells evidenced a 96.1%–100% genetic match with the ATCC reference.

To investigate the protective effects of CM from *APOA1/PON1* upregulated Huh7 cells upon TNFα-activated EC, the EA.hy926 cells were exposed to 10 ng/mL TNFα for 18 h, as detailed in our previous studies [37]. The medium was then removed and CM from transfected Huh7 was added in a 1:1 ratio with fresh RPMI, after the re-adjustment of D-glucose to 11 mM. After 24 h, the EC supernatant was collected, and cells were used for ROS measurements, monocyte adhesion evaluation, and WB analysis. The CM were collected from hepatocytes with upregulated *APOA1*, *PON1* or Control at similar confluence, and were normalized to the cellular protein measured by bicinchoninic acid (BCA) assay in cell lysates.

### Quantification of total ROS levels and monocyte adhesion to EC

Total ROS were determined in EC using 5 µM 2′,7′-dichlorodihydrofluorescein diacetate, after the exposure to TNFα followed by CM from transfected hepatocytes as described in the Supplemental Information. ROS levels normalized to total cellular protein content measured by BCA assay were presented as fold change of Control.

Monocyte adhesion to TNFα-activated EC exposed to CM from transfected hepatocytes was measured by using human THP-1 cells (ATCC, Manassas, USA) fluorescently labeled with 10 µM 2',7'-bis-(2-barboxyethyl)−5-(and-6)-barboxyfluorescein acetoxymethyl ester as described in the Supplemental Information. The adhesion of monocytes to EC was presented as fold change of Control.

### Design of experimental groups of animals

Twenty apoE^−/−^ male mice (B6.129P2-Apoetm1Unc/J; Stock No: 002052, Jackson Laboratory) aged 30 weeks were used for the experiment. All experimental procedures were done in accordance with the EU Directive 2010/63/EU and were conducted in compliance with the ARRIVE (Animal Research: Reporting of In Vivo Experiments) guidelines. The study was approved by the Ethics Committee of the ICBP-NS host institution (Approval no. 8/10.11.2022) and National Authority (Authorization no. 13/11.05.2023). The mice were divided into four groups (five per group) as follows: (1) mice receiving vehicle, 5% glucose solution (Sham), body weight (BW) 28.7 ± 0.5 g; (2) mice receiving control plasmids (Control), BW 29.8 ± 1.15 g; (3) mice receiving CRISPR/dCas9 plasmids for *Apoa1* activation (APOA1), BW 29.32 ± 1.35 g; and (4) mice receiving CRISPR/dCas9 plasmids for *Pon1* activation (PON1), BW 29.35 ± 1.05 g. Animals from each group were housed in two separate cages (2 + 3 animals). The randomization and exclusion criteria are detailed in the Supplemental Information. Plasmids (40 μg DNA/animal) were administered by injection in the tail vein, in a cocktail containing the in vivo-jetPEI® transfection reagent and 5% glucose solution (200μL total volume), according to the manufacturer's recommendation. The administration was done in a single dose, and after 30 days the animals were euthanized using 100 mg/kg ketamine and 15 mg/kg xylazine (i.p. injection). Sera, plasma and organs (liver, gall bladder, heart and aorta) were collected as described in the Supplemental Information. Two sets of feces samples were collected in the last week of the experiment from each cage and stored to −80 °C for cholesterol content analysis.

Serum TC level was measured by kit from Dialab, Neudorf, Austria. Serum HDL-C concentration was determined by the phosphotungstic acid/magnesium chloride precipitation method using Cholesterol HDL Precipitant (Dialab, Neudorf, Austria). APOA1 levels were measured by enzyme-linked immunosorbent assay (ELISA) (Mabtech, Nacka Strand, Sweden). Serum PON1 activity was measured as its ability to hydrolyze the paraoxon substrate, as described by Rozenberg et al. [44]. Levels of TBARS were measured in plasma using a method previously described [45]. Serum levels of TNFα and IL-10 were measured with kits from Mabtech (Nacka Strand, Sweden) and R&D Systems (MN, USA), respectively. To evaluate the potential detrimental effects of CRISPR/dCas9 plasmids administration in vivo, serum ALT and AST activities, and creatinine level were measured using commercially available kits (Dialab, Neudorf, Austria).

### Plasma lipoproteins analysis by FPLC

Pools (100 μl) from equal volumes of plasma from each group of mice were loaded onto a Superose 6/300 column in ÄKTApurifier system (GE Healthcare Life Sciences, United Kingdom) and eluted with PBS as described previously [46]. A total of 48 fractions of 500μL each were collected using Frac-920 fraction collector, and cholesterol content was measured as described above.

### Measurement of TC in the liver, bile and feces of mice

Two different samples of liver from each mouse were homogenized using the Hielscher Ultrasound Processor UP200S, in PBS, on ice. The obtained homogenates were centrifuged at 10,000xg, for 10 min at room temperature and TC was measured as described above. The obtained values were normalized to total protein level determined using the Lowry method [47]. The frozen gallbladders were allowed to thaw on ice, then punctured with a 25G sterile syringe needle, and subsequently centrifuged at 13,000xg for 5 min at 4^0^C. The released bile was collected and TC level was determined as described above. The lipids from mice’ feces were extracted and cholesterol was measured as previously described [36].

### Evaluation of lipid-deposits in thoracic aorta and plaque composition in aortic root of mice

The area of lipid deposits on the aortic arch and thoracic aorta was evaluated after Oil Red O staining [48], as described in Supplemental Information. The area of the lesions was measured using the ImageJ software (ImageJ, U. S. National Institutes of Health, Bethesda, Maryland, USA) and the results were normalized to the total surface of aortic arch and thoracic aorta. For evaluation of aortic root plaque composition, cryosections were stained with Nile Red for lipid identification, with Masson-Goldner’s trichrome for morphometric analysis, and were marked for CD68 and α-Smooth Muscle Actin (α-SMA) immunodetection. The nuclei were counterstained with DAPI as detailed in Supplemental Information. The antibodies used are listed in Table S2. Images were acquired using a Leica DMi8 fluorescence microscope (Leica Microsystems, Wetzlar, Germany) and processed with LAS X software (v3.7.4.23463), while maintaining identical acquisition parameters across channels and experimental groups. Nile Red-, CD68- and α-SMA-positive areas were quantified within manually defined vessel ROIs using ImageJ software. A constant threshold was applied to all samples for signal quantification. The Nile Red-, CD68- and α-SMA-positive areas expressed as the average percentage to the total area of aortic root lesions from 3–5 consecutive sections per animal. Brightfield images were captured by Olympus CKX41 inverted microscope (Olympus XC30 camera).

### RT-qPCR

Isolation of total RNA from cells and tissues was done with TRIzol reagent. Two μg of RNA were reverse-transcribed using the High-Capacity cDNA Reverse Transcription Kit. The obtained cDNA was amplified on a ViiA7 Real-Time PCR system (Applied Biosystems, MA, USA) using specific primers detailed in Table S1, and SyBr Select Master Mix. The mRNA levels was calculated using the “Fit Point Method” [49] relative to Control cells (for cells) or Sham (for mice), considered 1 unit.

### Western blotting analysis

Cells and liver tissue samples were homogenized on ice in RIPA lysis buffer supplemented with protease and phosphatase inhibitors, using Hielscher Ultrasound Processor UP200S. The homogenates were centrifuged at 10,000xg at 4 °C, the clear supernatants were collected, and the total protein levels were measured by BCA assay. Equal protein amounts (20–30 μg) were loaded on denaturant SDS-PAGE, and the expression of protein of interest was determined by WB technique using the specific antibodies from Table S2. The ratio of target protein to β-actin expression was determined by densitometric analysis of digital images obtained with an Amersham™ ImageQuant™ 800 analyzer and ImageQuant TL analysis software (Cytiva LifeSciences, MA, USA). Levels of secreted APOA1 and PON1 were evaluated in the CM of Huh7 cells by WB assay. The CM aliquots were thawed on ice, equal volumes (100 μl) were concentrated with trichloroacetic acid, and equal volumes (10 μl) of solubilized pellets were loaded on SDS-PAGE gels as described above. The levels of secreted APOA1 and PON1 were normalized to the total cellular protein measured by BCA assay. Protein expression was normalized relative to Control cells (for cells) or Sham (for mice), set to 1 unit. For APOA1 and PON1 quantification in FPLC fractions, equal volumes of HDL (20μL of fractions 30–36) or LDL (40µL of fractions 19–25) were concentrated by trichloroacetic acid precipitation, resuspended in 20µL of solubilization buffer, of which 10µL were loaded on SDS-PAGE, then the protein levels were evaluated by WB as described above. APOA1 or PON1 levels were expressed as the ratio of each fraction’s densitometric value to the corresponding one in Control group.

### Statistical analysis of the data

Statistical analysis and graphical representations of the study’s data were performed using *GraphPad Prism 10.0* (*GraphPad Software Inc*., San Diego, CA, USA). The Independent Student’s T-test was used to compare the data from APOA1 or PON1 group relative to Control. Statistical significance was established for *P* < 0.05. All results were presented as mean ± standard deviation (SD). Data from a minimum of three independent in vitro experiments were used for the statistical analysis, while each animal group consisted of five mice.

## Supplementary Information

Supplementary Material 1.

## Data Availability

The data that support the findings of this study are available from the corresponding author upon reasonable request.

## Abbreviations

APOA1: Apolipoprotein A1
apoE^−^^/^^−^: Apolipoprotein E-deficient
ALT/GPT: Alanine aminotransferase
AST/GOT: Aspartate aminotransferase
ABCG8: ATP-binding cassette sub-family G member 8 transporter
CVD: Cardiovascular diseases
CYP7A1: Cholesterol 7 alpha-hydroxylase
CRISPR/dCas9: Clustered regularly interspaced short palindromic repeats associated with deactivated endonuclease 9
CM: Conditioned medium
EC: Endothelial cells
FPLC: Fast protein liquid chromatography
HDL: High-density lipoproteins
HDL-C: High-density lipoprotein cholesterol
IDL: Intermediate-density lipoproteins
LXR: Liver X receptor
LDL: Low-density lipoproteins
MCP-1: Monocyte chemoattractant protein-1
PON1: Paraoxonase 1
RIPA: RadioImmunoPrecipitation Assay
RPL13a: Ribosomal Protein L13a
ROS: Reactive oxygen species
RCT: Reverse cholesterol transport
SCARB1: Scavenger receptor class B1
SIRT-1: Sirtuin 1
TBARS: Thiobarbituric acid-reactive species
TC: Total cholesterol
TNFα: Tumor necrosis factor α
VCAM-1: Vascular cell adhesion molecule-1
