# Supplementary material for: CRISPR/dCas9-induced upregulation of endogenous apolipoprotein A1 and paraoxonase 1 genes reduces the aortic lipid deposits in apoE−/− mice
Source: Mol Biomed. 2026 Sep 18;7:176. doi: 10.1186/s43556-026-00580-8 (PMC13586135; doi:10.1186/s43556-026-00580-8)
Supplement: Supplementary file 1 [file 43556_2026_580_MOESM1_ESM.docx]

**MBMC-D-26-00114R3**

**Supplemental Information**

**CRISPR/dCas9-induced upregulation of endogenous apolipoprotein A1 and paraoxonase 1 genes reduces the aortic lipid deposits in apoE^-/-^ mice**

**Authors**

Laura Toma^#1^, Teodora Barbălată^#1^, Jessica Isabela Cătălina Hărătău^1^, Gabriela Maria Sanda^1^, Elena Valeria Fuior^2^, Ioana Mădălina Fenyo^2^, Loredan Ştefan Niculescu^1^, Laura-Maria Dăian^3^, Shlomo Sasson^4^, Anca Volumnia Sima^1^, Camelia Sorina Stancu^1*^

^1^Lipidomics Department, Institute of Cellular Biology and Pathology “Nicolae Simionescu” of the Romanian Academy, 8, B.P. Haşdeu Street, 050568 Bucharest, Romania,

^2^Gene Regulation and Molecular Therapies Laboratory, Institute of Cellular Biology and Pathology “Nicolae Simionescu” of the Romanian Academy, 8, B.P. Haşdeu Street, 050568 Bucharest, Romania

^3^BetaUpreg Research Group, Institute of Cellular Biology and Pathology “Nicolae Simionescu” of the Romanian Academy, 8, B.P. Haşdeu Street, 050568 Bucharest, Romania

^4^Department of Pharmacology, Institute for Drug Research, Faculty of Medicine, The Hebrew University of Jerusalem, 9112002 Jerusalem, Israel

^#^These authors contributed equally to this work.

^*^Corresponding author: Camelia Sorina Stancu, PhD,

E-mail: [camelia.stancu@icbp.ro](mailto:camelia.stancu@icbp.ro)

**Supplemental Figures**

**Fig. S1. Cellular reactive oxygen species (ROS) production expressed relative to cellular protein (a) and THP-1 monocytes adhesion to activated EC (b).** TNFα-activated EC were exposed to conditioned media (CM) from un-transfected Huh7 cells (Control Hep) or transfected with Control plasmids. All data are expressed as fold change compared to un-transfected and not exposed to TNFα EC, and presented as mean ± SD of 3 independent experiments. **p<0.01, ***p<0.001 versus un-transfected EC exposed to TNFα.

**Fig. S2. Parameters indicating innate immune response in apoE^-/-^ mice injected with CRISPR/dCas9 system for *Apoa1* and *Pon1*.** Liver gene expression of interferon beta 1 (*Ifnb1*) (a) and interleukin-6 (*Il6*) (b), IL-6 protein levels in the liver of mice from APOA1 group (c) or PON1 group (d) and in serum (e). All data are expressed as fold change compared to Sham group, and presented as mean ± SD of 3-5 animals.

**Fig. S3** **PON1 level in the FPLC fractions separated from the plasma of apoE^-/-^ mice injected with CRISPR/dCas9 plasmids for *Pon1* activation.** HDL-bound PON1 (fractions no. 30-33) (a) and free PON1 (fractions no. 34-36) (b) levels in the FPLC fractions from PON1 mice as compared to Control group. All data are expressed as fold change relative to the equivalent fraction of the Control group and presented as mean ± SD (3-4 fractions from pool of 5 plasma).

**Supplemental Material and Methods**

**Reagents**

RPMI-1640 medium (RPMI), Dulbecco’s Modified Eagle’s Medium (DMEM), penicillin, streptomycin, neomycin, 2′,7′-Dichlorofluorescein diacetate, (DCFH-DA), 2',7'-Bis-(2-Carboxyethyl)-5-(and-6)-Carboxyfluorescein, Acetoxymethyl Ester (BCECF-AM), Oil Red O, protease inhibitor cocktail, sodium fluoride and sodium orthovanadate, bicinchoninic acid solution (BCA) were from Sigma-Aldrich Co. (MO, USA). Masson-Goldner’s trichrome staining kit was from Carl Roth (Karlsruhe, Germany). Tumor necrosis factor α (TNFα) (210-TA-020/CF) was from R&D Systems (MN, USA), and ECL chemiluminescent substrate from AppliChem GmbH (Darmstadt, Germany). Fetal bovine serum (FBS) was from Euroclone, EU. *In vivo*-JetPEI^®^ was from Polyplus (Illkirch, France), TRIzol reagent, Nile Red and DAPI were from Invitrogen, Thermo Fisher Scientific (MA, USA). SyBr Select Master Mix and High-Capacity cDNA Reverse Transcription Kit were from Applied Biosystems (MA, USA). Human *APOA1* (sc-400499-ACT), human *PON1* (sc-402701-ACT), mouse *Apoa1* (sc-419158-ACT), mouse *Pon1* (sc-422343-ACT) and Control (sc-437275) CRISPR/dCas9 activation plasmids, transfection medium (sc-108062), and UltraCruz Transfection Reagent (sc-395739) were from Santa Cruz Biotechnology (CA, USA). The CRISPR Activation Plasmids are licensed products of Santa Cruz Biotechnology, Inc., and permit the identification and upregulation of specific genes by utilizing a D10A and N863A deactivated Cas9 (dCas9) nuclease fused to a VP64 activation domain, in conjunction with sgRNA (MS2), a target-specific sgRNA engineered to bind the MS2-P65-HSF1 fusion protein. All these components constitute a synergistic activation mediator (SAM) transcription activation system. Human *APOA1*, human *PON1*, mouse *Apoa1*, and mouse *Pon1* CRISPR Activation Plasmid are each a SAM transcription activation system designed to specifically upregulate expression of the *APOA1* human gene (mapping to 11q23.3), *PON1* human gene (mapping to 7q21.3), *Apoa1* mouse gene (mapping to 9 A5.2) and *Pon1* mouse gene (mapping to 6 A1). Each SAM transcription activation system consists of the following 3 plasmids at a 1:1:1 mass ratio: the CRISPR/dCas9-VP64-Blast plasmid encoding the deactivated Cas9 (dCas9) nuclease (D10A and N863A) fused to the transactivation domain VP64, and a blasticidin resistance gene; the MS2-P65-HSF1-Hygro plasmid encoding the MS2-p65-HSF1 fusion protein, and a hygromycin resistance gene; the sgRNA (MS2)-Puro plasmid encoding a target-specific 20 nt guide RNA, and a puromycin resistance gene. The sgRNA (MS2)-Puro plasmids in apoA1 and PON1 CRISPR Activation Plasmids (human and mouse) each encode their own, unique, target-specific 20 nt guide RNA.

**Cell culture**

Hepatocytes from human hepatocarcinoma (Huh7 transformed cell line, Cell Lines Service GmbH, Germany), were grown in RPMI-1640 supplemented with fetal bovine serum (FBS) (10%, v/v), penicillin (100 U/mL), and streptomycin (0.1 mg/mL).

Human umbilical vein EC (EA.hy926 transformed cell line, ATCC, Manassas, VA, USA), mycoplasma free, were grown in DMEM supplemented with 10% FBS, 0.1 mg/mL/100 U/mL streptomycin/penicillin, and 50 µg/mL neomycin.

**Determination of total ROS levels in ECs**

For the detection of intracellular ROS levels, the cells were loaded with 5µM DCFH-DA, at 37°C. After 30min, the excess of DCFH-DA was washed with warm culture medium, and the fluorescence of ROS-sensitive fluorophore (Ex=485nm/Em=535nm) was measured in whole cells (Tecan Infinite M200 spectrofluorometer, Tecan, Austria). The fluorescence values were normalized to protein concentration measured by bicinchoninic acid (BCA) assay.

**Quantification of monocyte adhesion to ECs.**

On brief, the THP-1 human monocytes (ATCC, Manassas, VA, USA) were loaded with 10μmol/L BCECF-AM fluorochrome at 37°C. After 30min, the BCECF-AM in excess was washed by centrifugation, and the fluorescently-labelled THP-1 cells were interacted with the ECs at 37°C. After 30min, the non-adhered THP-1 cells were gently removed by washing with RPMI and the adhered THP-1 cells were lysed in a solution containing 1% Triton X‐100 and 0.1 M NaOH. The BCECF fluorescence, proportional to the adhered THP-1 cells, was measured at 485nm (Ex)/535nm (Em).

**Design of experimental groups of animals; collection of sera/plasma and organs from mice**

The mice were bred and housed in the specific pathogen-free (SPF) animal facility of the host Institute, in ventilated cages, at 24°C constant ambient temperature, with alternating cycles of dark/light (12h/12h), and water and standard food provided ad libitum.

We applied randomization during animal assignation to the groups, by matching mice by litter and body weight, and on course of the experiment (care and treatment). We used numerical codes for animals and cages, and the group allocation was concealed from animal care staff, the researcher assigned to administrate the activation plasmids and from part of the researchers performing the assays and analyzing data. The exclusion criteria were: female gender, more than three days after birth, and over 2 g differences in BW. The sample size for each group was calculated using the online tool available at <https://clincalc.com/stats/samplesize.aspx>.

After the treatment and euthanasia, sera, plasma and organs (liver, gall bladder, heart and aorta) were collected as follows. The anesthetized animals were subjected to laparotomy, and blood was collected from heart in *BD Microtainer (SST Tubes)* (for serum) and *BD Microtainer (K2E Tubes)* (for plasma) (*BD*, Franklin Lakes, New Jersey, USA). The sera and plasma samples were aliquoted and stored at −80°C until use. The mice vasculature was perfused with phosphate saline buffer (PBS) to remove the blood. The gall bladder was carefully harvested and stored at -80^o^C until analysis. Collected livers were weighed and cut into small pieces, then snap-frozen in liquid nitrogen and kept at −80°C until use. The aortas were collected, kept in 4% paraformaldehyde (PFA) at 4°C, and processed for Oil Red O staining of the atheroma.

**Evaluation of aortic lipid-deposits in mice**

In brief, after fixation in PFA, the specimens were washed in PBS, the remaining adipose tissue was carefully removed, the arteries were cut along the longitudinal axis, and kept in PBS during pin fixation for *en face* exposure. The aortic lesions were stained by incubation with a 0.3% Oil Red O solution in 60% isopropanol, for 50min, in the dark. The aortas were briefly rinsed 3-times in 60% isopropanol to remove the Oil Red O excess and left in distilled water for stereomicroscope visualization and photography (Stemi 2000C microscope, AxioVision Rel. 4.8 software, Carl Zeiss Microscopy GmbH or LLC, NY USA). The area of the lesions was measured using the ImageJ software (ImageJ, U. S. National Institutes of Health, Bethesda, Maryland, USA) and the results were normalized to the total surface of aortic arch and thoracic aorta.

**Evaluation of aortic root plaque composition**

Cryosections of aortic root (5 µm thick) were stained with Nile Red (Invitrogen-Molecular Probes, Eugene, OR, USA) for lipid identification. For identification of plaque composition, the cryosections were marked for CD68 and α-Smooth Muscle Actin (see Table S2 for antibodies used). The cryosections were permeabilized with 0.5% Triton X-100 for 20 min and blocked with 1% BSA 30 min at room temperature. Primary antibodies were diluted in 1% BSA and applied for 1h at room temperature, followed by secondary antibodies for 45 min. The nuclei were counterstained with DAPI (ThermoFisher Scientific). Images were acquired using a Leica DMi8 fluorescence microscope (Leica Microsystems, Wetzlar, Germany) and processed with LAS X software (v3.7.4.23463), while maintaining identical acquisition parameters across channels and experimental groups. Nile Red-positive area and protein expression of CD68 (red fluorescence) and α-SMA (green fluorescence) were quantified within manually defined vessel ROIs using ImageJ software. A constant threshold was applied to all samples for signal quantification. Lipid accumulation was expressed as average percentage of Nile Red-positive area to the total aortic wall area from 3-5 consecutive sections per sample. For morphometric analysis of the aortic root lesions, the cryosections were stained with Masson-Goldner’s trichrome staining kit (Roth # 3459.1) following the manufacturer’s instructions. Brightfield images were captured using an Olympus CKX41 inverted microscope with an Olympus XC30 camera. The inner vessel border (luminal surface) and the outer border of the aortic wall were manually delineated on cross-sectional images using ImageJ software. The lesion area was defined as abnormal intimal tissue extending into the vascular lumen. Lesion burden was expressed as the average percentage of lesion area relative to the total aortic wall area from 3-5 consecutive sections per sample.

**Table S1. Sequence of primers used for Real-Time PCR**

| **Gene** | **GeneBank accession number** | **Sequences of oligonucleotide primers** |
| --- | --- | --- |
| **hAPOA1** | NM_000039.2 | FW: 5’-CCCTGGGATCGAGTGAAGGA-3’  RV: 5'-CTGGGACACATAGTCTCTGCC-3’ |
| **hPON1** | NM_000446.5 | FW: 5’-CTATGACTCAGAGAATCCTCCTGCATCAG-3’  RV: 5'-CATGGGTGCAAATCGGTCTGTTAGAGC-3’ |
| **hRPL13A** | NM_001101.3 | FW: 5'-GTCTTCCCCTCCATCGT-3'  RV: 5'-CGTCGCCCACATAGGAAT-3' |
| **mApoa1** | NM_009692.4 | FW: 5'-GGCAGAGACTATGTGTCCCAGT-3’  RV: 5’-GCTGACTAACGGTTGAACCCAG-3’ |
| **mPon1** | NM_011134.4 | FW: 5'-GGTGTTGGCACTTTACAAGAACC-3’  RV: 5’-GGCGTTACTTCACGGAAAGC-3’ |
| **mScarb1** | NM_016741.3 | FW: 5'-ACACCCGAATCCTCGCTGGAAT-3’  RV: 5’-CCGTTGGCAAACAGAGTATCGG-3’ |
| **mAbcg8** | NM_026180.3 | FW: 5'-GGTCCTTCTGATGACATCTGGC-3’  RV: 5’-CGTCTGTCGATGCTGGTCAAGT-3’ |
| **mCyp7a1** | NM_007824.3 | FW: 5’-CACCATTCCTGCAACCTTCTGG-3’  RV: 5’-ATGGCATTCCCTCCAGAGCTGA-3’ |
| **mIl6** | NM_031168.2 | FW: 5’-TACCACTTCACAAGTCGGAGGC-3’  RV: 5’-CTGCAAGTGCATCATCGTTGTTC-3’ |
| **mIfnb1** | NM_010510.2 | FW: 5’-GCCTTTGCCATCCAAGAGATGC-3’  RV: 5’-ACACTGTCTGCTGGTGGAGTTC-3’ |
| **mRpl13a** | NM_009438.5 | FW: 5'- CTGCTCTCAAGGTTGTTCGGCT -3’  RV: 5’- CCTTCCGTTTCTCCTCCAGAGT -3’ |

**Table S2.** **Antibodies used for Western blotting and immunohistochemistry analysis**

|  | **Antibody** | **Specificity** | **Catalogue #** | **Dilution** | **Source** |
| --- | --- | --- | --- | --- | --- |
|  | Mouse mAb | ApoA-I | sc- 376818 | 1:400 | Santa Cruz |
|  | Rabbit mAb | PON1 | ab126597 | 1:3000 | Abcam |
|  | Rabbit Polyclonal | ABCG8 | sc-30111 | 1:500 | Santa Cruz |
|  | Rabbit mAb | SR-B1 | ab217318 | 1:4000 | Abcam |
|  | Mouse mAb | Sirt-1 | sc-74504 | 1:600 | Santa Cruz |
|  | Mouse mAb | VCAM-1 | ab134047 | 1:2000 | Abcam |
|  | Rabbit Polyclonal | MCP-1 | ab9669 | 1:2000 | Abcam |
|  | Mouse mAb | β-actin | sc-47778 | 1:2000 | Santa Cruz |
|  |  | Rabbit Anti-Mouse IgG H&L (HRP) | ab6728 | 1:10000 | Abcam |
|  |  | Goat Anti-Rabbit IgG H&L (HRP) | ab6721 | 1:10000 | Abcam |
|  | Rabbit mAb | CD68 | MA5-56511 | 1:200 | Invitrogen |
| Mouse mAb | | α-Smooth Muscle Actin | 14-9760-82 | 1:200 | Invitrogen |
|  | | Donkey anti-rabbit AF568 | A10042 | 1:200 | Invitrogen |
|  | | Goat anti-mouse AF488 | A11001 | 1:200 | Invitrogen |
